# Understanding the Controlling Factors for CO_2_ Sequestration in Depleted Shale Reservoirs Using Data Analytics and Machine Learning

**DOI:** 10.1021/acsomega.2c01445

**Published:** 2022-06-07

**Authors:** Hassan Khaled Hassan Baabbad, Emre Artun, Burak Kulga

**Affiliations:** † Department of Environment, Land and Infrastructure Engineering, Politecnico di Torino, 10129 Torino, Italy; ‡ Department of Petroleum and Natural Gas Engineering, Istanbul Technical University, Maslak, 34469 Sariyer, Istanbul, Turkey

## Abstract

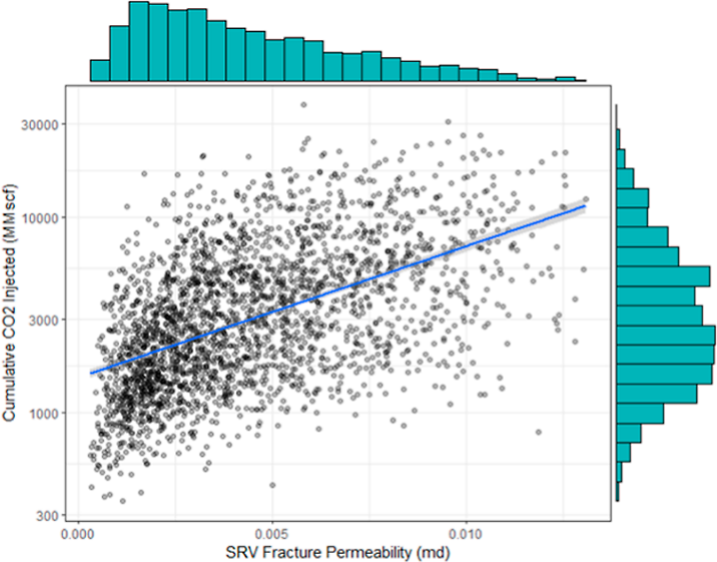

Carbon capture and sequestration is the process of capturing carbon dioxide (CO_2_) from refineries, industrial facilities, and major point sources such as power plants and storing the CO_2_ in subsurface formations. Carbon capture and sequestration has the potential to generate an industry comparable to, if not greater than, the existing oil and gas sector. Subsurface formations such as unconventional oil and gas reservoirs can store significant quantities of CO_2_. Despite their importance in the oil and gas industry, our understanding of CO_2_ sequestration in unconventional reservoirs still needs to be developed. The objective of this paper was to use an extensive data set of numerical simulation results combined with data analytics and machine learning to identify the key parameters that affect CO_2_ sequestration in depleted shale reservoirs. Machine learning-based predictive models based on multiple linear regression, regression tree, bagging, random forest, and gradient boosting were built to predict the cumulative CO_2_ injected. Variable importance was carried out to identify and rank important reservoir and operational parameters. The results showed that random forest provided the best predictive ability among the machine learning techniques and that regression tree had the worst predictive ability, mainly because of overfitting. The most significant variable for predicting cumulative CO_2_ sequestration was stimulated reservoir volume fracture permeability. The workflows, machine learning models, and results reported in this study provide insights for exploration and production companies interested in quantifying CO_2_ sequestration performance in shale reservoirs.

## Introduction

Carbon capture and sequestration has received worldwide attention as a potential technique for reducing carbon dioxide (CO_2_) emissions into the atmosphere and is therefore one approach for mitigating climate change. Target geologic reservoirs for CO_2_ sequestration include saline aquifers, and both conventional and unconventional oil and gas reservoirs. Unconventional reservoirs (such as shale reservoirs) are rapidly becoming the leading source of energy.^1^ Owing to their large application scale (wide area and size), structure, and fracture network generated by hydraulic fracturing, unconventional reservoirs have lately been identified as valuable target geologic reservoirs for CO_2_ sequestration.^2^ Modeling studies are currently being conducted to investigate the uncertainties associated with storing CO_2_ in shale reservoirs and the associated economic and sustainability characteristics of these targets.^2^ This study uses an extensive data set of numerical simulation results combined with data analytics and machine learning to identify the key parameters that affect CO_2_ sequestration in depleted shale reservoirs.

Standard industry practice is to model CO_2_ geologic sequestration using laboratory experiments and physics-based simulators. For example, in the Barrow Sub-basin field in Western Australia, reservoir modeling was used to predict the behavior of CO_2_ in the reservoir in response to CO_2_ injection. The researchers discovered that the direction of CO_2_ movement and geological structure were two important factors to consider when choosing the best well configuration. This satisfied the injection condition while also confirming the feasibility of CO_2_ geological sequestration in the Barrow Sub-basin.^3^ Furthermore, a numerical reservoir simulator (PSU-SHALECOMP) was used for testing CO_2_ sequestration in depleted shale formations.^4^ Their analysis showed that hydraulically fractured horizontal wells in shale reservoirs are ideal candidates for CO_2_ sequestration because of their ultra-tight features, which make them a promising source for safely storing CO_2_ over geologic time with better injection and production design. In addition, a numerical simulator STOMP (Subsurface Transport Over Multiple Phases) was used to examine the effect of Mount Simon’s well spacing, injection depth, and reservoir properties for CO_2_ geologic sequestration. The research demonstrated that Mount Simon has enough injectivity to meet the desired CO_2_ storage levels. The study also showed that a well-designed set of 2D single-well simulations may investigate the trade-off between target injection volume and well estimate.^5^ Nonetheless, the issue with physics-based simulators is the computational effort needed. Previous researchers were able to point out that the main problem with numerical reservoir simulation is the high computational cost and long run times.^6,7^ This is owing to the requirement to run a huge number of simulations to support practical decisions including production optimization, optimal well spacing, and field development.^7^

In the meantime, data analytics and machine learning have been proposed to understand the behavior of CO_2_ sequestration in saline aquifers and conventional and unconventional reservoirs, after advancements in digital transformation in the oil and gas industry. Data analytics is the process of evaluating data, understanding what it says, learning from it, and creating predictions based on these data-driven insights.^8^ Whereas machine learning is the method of constructing a model between input and output variables by using an algorithm to determine the underlying independent and dependent relationship from data.^9^ Several papers in recent years have focused on the use of data analytics and machine learning to assess unconventional reserves.^1,7,10^ However, these papers mostly concentrated on production optimization in unconventional reserves. Consequently, there are many questions that arise regarding the operational aspects of CO_2_ sequestration in unconventional reservoirs. For example, what are the typical characteristics of a high-volume sequestration scenario? Also, what causes low-performance or high-performance CO_2_ sequestration process? In this paper, we aim to find solutions to these concerns through a data analytics and machine learning approach by analyzing a large set of numerical simulation scenarios. The fundamental objectives of this study are:

1. Discovering hidden trends and patterns by conducting an exploratory data analysis over the collected data.
2. Analyzing the impact of reservoir and operational parameters on the cumulative CO_2_ injected using correlation coefficients.
3. Development of predictive models based on machine learning that can predict the performance of CO_2_ sequestration process given the set of operational and reservoir parameters.
4. Investigating the main influence of operational and reservoir parameters on the CO_2_ sequestration performance via variable importance analysis.

The first two objectives are achieved by applying data analytics to the results obtained from numerical simulation scenarios. Histograms of reservoir and operational parameters, as well as scatterplots of reservoir and operational parameters versus cumulative CO_2_ injected, and correlation coefficients are used to complete the exploratory data analysis. Subsequently, machine learning-based predictive models, for instance, based on multiple linear regression, tree methods such as regression, bagging, random forest, and gradient boosting are implemented. These predictive models are trained using the data set, which take the reservoir and operational parameters as the main predictors and predict the cumulative CO_2_ injected as the main response variable. In the following section, the methods used to attain these objectives are described. The approach is followed by the results and discussion, as well as the main conclusions.

## Methodology

This research presents a novel approach to identifying what drives the low-performance or high-performance CO_2_ sequestration process in shale gas reservoirs by implementing a workflow that uses data analytics procedures and machine learning algorithms. This methodology not only produces effectively accurate results but also is less time-consuming considering that the time spent on building the predictive models and performing a variable importance is comparatively small.

### Simulator and Data Set

**Figure 1:**
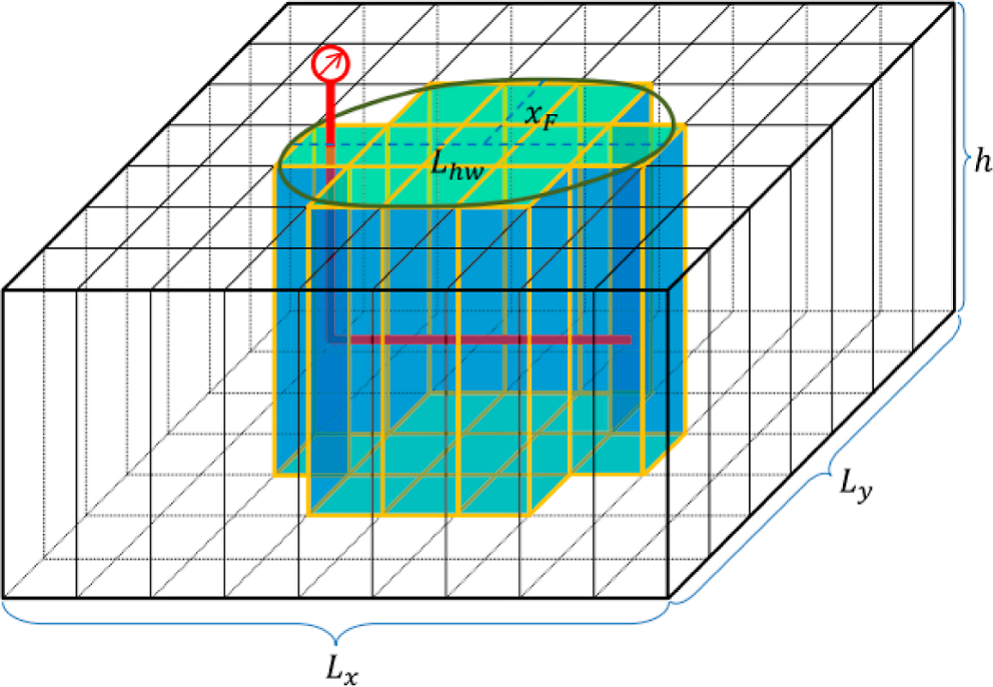
SRV technique used to approximate the induced fracture network in the numerical model.^4^ Republished with permission of Elsevier Science & Technology Journals, from Kulga, B. and Ertekin, T. Numerical representation of multi-component gas flow in stimulated shale reservoirs. 2018, **56**; permission conveyed through Copyright Clearance Center, Inc.

In this study, the data set used consisted of a considerable number of numerical simulation scenarios (approximately 1400) that were conducted in a computational simulator as part of another research study.^11^ The reservoir model utilized was a Penn State University-developed compositional dual-porosity, dual-permeability, and multi-phase reservoir simulator known as PSU-SHALECOMP. The network of induced fractures is described in the numerical model using the stimulated reservoir volume (SRV) technique, which approximates the fracture network as an elliptical region (Figure 1) around the horizontal well. The PSU-SHALECOMP simulator takes into account the influence of water as well as the swelling and shrinking of the matrix.^4^ After a main gas recovery phase, CO_2_ sequestration was undertaken with a constant injection rate limitation until a predefined fracturing pressure limit was achieved in these simulations.^2^

The full data set constituted 22 predictor variables and two response variables. However, in this paper only 18 predictor variables were considered and one response variable. For each variable, pre-specified ranges were utilized to produce uniformly distributed scenarios.^11^ A specific numerical simulation scenario where the volume of sequestered CO_2_ is collected consists of a combination of input variables.^2^ Predictor variables consist of reservoir parameters and operational parameters. The response variable is the cumulative CO_2_ injected (MMscf). All the variables in the data set had non-missing values for the cumulative CO_2_ injected. Table 1 shows the list of all the variables used in this paper and their corresponding variable names.

**Table 1:** List of Variables Used

description	variable	type
cumulative CO_2_ injected (MMscf)	cum_inj	response
thickness (ft)	Thickness	predictor
matrix porosity (%)	PoroM	predictor
fracture porosity (%)	PoroF	predictor
water saturation in the matrix (%)	WatSatM	predictor
matrix permeability (md)	PermM	predictor
fracture permeability (md)	PermF	predictor
fracture spacing (ft)	Xs	predictor
initial pressure (psi)	InitPres	predictor
initial temperature (F)	InitTemp	predictor
horizontal wellbore length (ft)	LHW	predictor
hydraulic fracture length (ft)	Lf	predictor
length of reservoir in *x* direction (ft)	edge_x	predictor
length of reservoir in *y* direction (ft)	edge_y	predictor
SRV fracture porosity (%)	SRV_phi_f	predictor
SRV fracture permeability (md)	SRV_kf	predictor
SRV fracture spacing (ft)	SRV_xs	predictor
total production time (days)	TimeProd_Total	predictor
fracture pressure (psi)	Pfrac	predictor

### Exploratory Data Analysis

The first part of our methodology involved performing an exploratory data analysis. The main aim of any exploratory data analysis study is to maximize the understanding of the data set under consideration.^12^ Understanding the data set implies identifying and revealing the underlying structure of the data.^12^ Exploratory data analysis can be categorized into (a) univariate data analysis, (b) bivariate data analysis, and (c) multivariate data analysis. In this study, graphical techniques of exploratory data analysis were mainly utilized to ascertain patterns, features, and correlations in the shale reservoir data set. The exploratory data analysis graphing techniques used in this paper include:

1. Univariate graphing using histograms;
2. Bivariate graphing using scatterplots; and
3. Multivariate graphing using a correlation matrix.

#### Univariate Graphing

In analyzing our data set because the variables had ranges of values, we can then explore the distribution of the data by using histograms. The histogram is the most common univariate tool for the display of continuous values.^1^ The data density is visually represented by a histogram.^12^ It is created by dividing the observed range into multiple intervals (bins) and then plotting the actual frequency of occurrence within each interval.^8^ In most cases, the number of bins used in histograms is determined through trial and error.^8^

#### Bivariate Graphing

A scatterplot is the most useful graph to show the relation between two quantitative variables.^13^ In this study, scatterplots were used to display and analyze the relationship between the predictor variables and the main response variable. The values of predictor variables will appear on the *x*-axis, whereas the values of the primary response variable will be shown on the *y*-axis. The absolute value of the correlation coefficient determines the strength of a linear relationship.^8^ The following equation can be used to calculate the Pearson correlation coefficient^8^
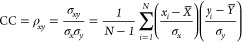
1where σ_*x*_ is the standard deviation of *x*, σ_*y*_ is the standard deviation of *y,* σ_*xy*_ is the covariance, *X̅* is then mean of *x,Y̅* is the mean of *y*, *N* – 1 is the degree of freedom, *x*_i_ is the individual outcome for *x ,*and *y*_*i*_ is the individual outcome for *y.*

One important aspect about the correlation coefficient is that it pertains to a monotonic relationship.^8^ Moreover, the linear assumption of the Pearson correlation may not apply to nonlinear predictor-response relationships mainly because the Pearson correlation considers sample data which is from a bivariate normal distribution.^14^

#### Multivariate Graphing

After visualizing the data using scatterplots, we analyzed the strength of the linear association between the predictor variables and the response variables using the correlation matrix. This implies that the calculation and display of the correlation coefficient for all pairs of variables is the correlation matrix.^8^

### Predictive Modeling

In this study, the response variable (target variable) for all predictive modeling was cumulative CO_2_ injected and the predictor variables for all predictive modeling were the 18 reservoir and operational parameters presented in Table 1. Predictive modeling is the process of creating a tool or a model that allows for accurate predictions.^10^ The main emphasis of this section is on predictive statistical modeling, in which statistical and machine learning techniques were used to determine the dependence or relationship between response and predictor variables. In this study, two main techniques were used:

1. Multiple linear regression and
2. Tree-based methods (regression tree, bagging, random forests, and boosting).

#### Multiple Linear Regression

To predict the volume of CO_2_ sequestered, multiple linear regression^15,16^ was applied between the response variable (cumulative CO_2_ injected) and predictor variables (reservoir parameters and operational parameters). Multiple linear regression was used because we had multiple predictor variables of reservoir and operational parameters. After determining the regression coefficients, we can quantify the model fit or the amount of variability explained by the multiple linear regression model using the *R*^2^. *R*^2^ is the square of the correlation between the response and the fitted linear model in multiple linear regression; in fact, one quality of the fitted linear model is that it optimizes this correlation among all feasible linear models.^15^ The model explains a significant proportion of the response variable variation if the *R*^2^ value is closer to 1.

Another metric which can be used to evaluate the model fit is the mean squared error (MSE). Rather than the absolute value, MSE calculates the average squared difference between the actual value and the prediction.^7^ The MSE can be calculated by the following equation^7^
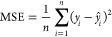
2

The MSE is evaluated by the squared units of the response variable.^7^ Numbers closer to zero are preferred, as they suggest smaller discrepancy between the actual and predicted values.^7^ Because of its well-known distribution qualities, such as being continuously differentiable and being an adequate statistic for normally distributed processes, MSE is often selected relative to average absolute error (AAE).^7^ The multiple linear regression modeling was completed using the caret library in R.^16,17^

#### Regression Tree

In the second approach for predictive modeling, the regression tree machine learning algorithm was used. For regression tree, the algorithm should decide to automatically divide the variables and split points, as well as the topology (form) of the tree.^18^ The regression tree modeling was completed using the tree library^19^ in R^16^.

#### Bagging

Bootstrap or bagging is a broad strategy to reduce the variance of a statistical learning method. We have included it here because it is very beneficial and common in the context of decision trees.^15^ Taking several training sets from the population, building a different prediction model using each training set, then averaging the resulting predictions is a natural approach to minimize variance and hence raises the prediction accuracy of a statistical learning method.^15^ The bagging technique was completed using the randomForest library^20^ in R^17^.

#### Random Forests

Random forests^21^ machine learning algorithm is a significant variation of bagging in which a considerable number of de-correlated trees are built and then averaged.^18^ By definition, a random forest is a type of classifier which consists of a collection of trees categorized by classifiers {*h*(**x**,Θ_*k*_), *k* = 1, ...}, each tree casts a unit vote for the most popular class at input **x**, and the {Θ_*k*_} are independent identically distributed random vectors.^21^

For regression, random forests are created by developing trees based on a random vector Θ, so that the tree predictor *h*(**x**,Θ) takes on numerical values rather than class labels.^21^ We assume that the training set is drawn separately from the distribution of the random vector ***Y*** and ***X*** and that the output values are numerical.^21^ The random forests machine learning algorithm was completed using the randomForest library^20^ in R^17^.

#### Boosting

Boosting was the final ensemble tree method used for predictive modeling. Boosting is a comprehensive method that refers to a variety of statistical learning approaches for regression and classification.^15^ Boosting is identical to bagging, except that the trees are built sequentially, which means that each tree is grown using information from prior trees.^15^ Instead of bootstrap sampling, boosting uses a changed version of the original data set to suit each tree.^15^ The boosting machine learning algorithm was completed using the gbm library^22^ in R^17^.

#### Hyperparameter Tuning

Hyperparameter tuning is a critical component of the overall modeling process to prevent overfitting and yield the best model. Gradient boosting, random forest, and neural networks are examples of machine learning techniques for regression and classification that require a set of hyperparameters to be tuned before they can be used.^23^ Machine learning practitioners can utilize default values of hyperparameters defined in softwares or manually set them up, for instance, based on recommendations from the literature, experience, or trial-and-error, to select an appropriate hyperparameter configuration for a particular data set at hand.^23^

The tree-based methods discussed earlier contain a set of hyperparameters. For decision tree method, some of the hyperparameters include maximum depth, minimum samples split, minimum samples leaf, maximum features, and minimum impurity decrease.^24^ The maximum depth is a hyperparameter in which during the training phase it determines the maximum level to which a tree can go down.^24^ The second hyperparameter minimum samples split enables the user to control how many samples a node must have to be splitable.^24^ A similar hyperparameter to minimum samples split is called minimum samples leaf which limits the number of situations that a terminal leaf node can contain.^24^ Moreover, the maximum features is used to prevent overfitting. Through selecting a smaller number of features, we may improve the tree’s stability while reducing variability and overfitting.^25^ Lastly, the minimum impurity decrease hyperparameter allows us to determine how deep our tree grows in relation to the impurity level.^25^ Post-processing pruning technique is another option for avoiding overfitting in decision trees (regression trees).^26^ Hence, in this paper to determine the ideal level of our regression tree complexity, we utilized cross-validation together with cost complexity pruning to choose a series of trees to evaluate.

Following regression tree, the next method which contained a set of hyperparameters is random forest. One of the most fundamental hyperparameters of the random forest algorithm is the *mtry*.^27^ This hyperparameter randomly picks candidate variables from which each split is taken when constructing a tree. Conventionally, in various software programs, *mtry* is set to  for classification problems and  for regression problems with *p* being the number of predictor variables.^27^ Another hyperparameter in the random forest algorithm is the node size. This hyperparameter controls the minimum number of observations in a terminal node.^27^ Other hyperparameters for random forest include number of trees and splitting rule.^27^ In this study, the main hyperparameter we considered was the *mtry*. To tune this hyperparameter, we used the tuneRF from the randomForest package. This procedure provided the best *mtry* value based on out-of-bag error.

Lastly, hyperparameter tuning was performed for the boosting algorithm. In this algorithm, there are mainly three tuning parameters. The number of trees, shrinkage parameter (λ), and number *d* of splits.^15^ The shrinkage parameter determines how quickly the boosting algorithm learns and the complexity of the boosting algorithm is regulated by number *d* of splits.^15^ In this paper, in order to tune the hyperparameters of the boosting algorithm, we used default values as defined in the software.

#### Variable Importance

In this study, variable importance was used to identify which of the 18 predictor variables (reservoir and operational parameters) were most significant in the predictive model for estimating the response variable (cumulative CO_2_ injected). Two main approaches were utilized which were random forests and gradient boosting machines (GBMs). The prediction effectiveness of each variable in random forests is determined by calculating the increase in MSE when that parameter is modified, whereas the others are kept unchanged.^7^ The principle behind the arrangement stage is that, if the input parameter is not significant, readjusting its values among the training observations will not make a substantial difference in the model’s prediction accuracy.^7^ Likewise, the variable importance in GBMs is determined by the number of times a predictor variable is split, weighted by the squared improvement in the model because of each split, and averaged over all trees.^7^

## Results and Discussion

### Histograms

**Figure 2:**
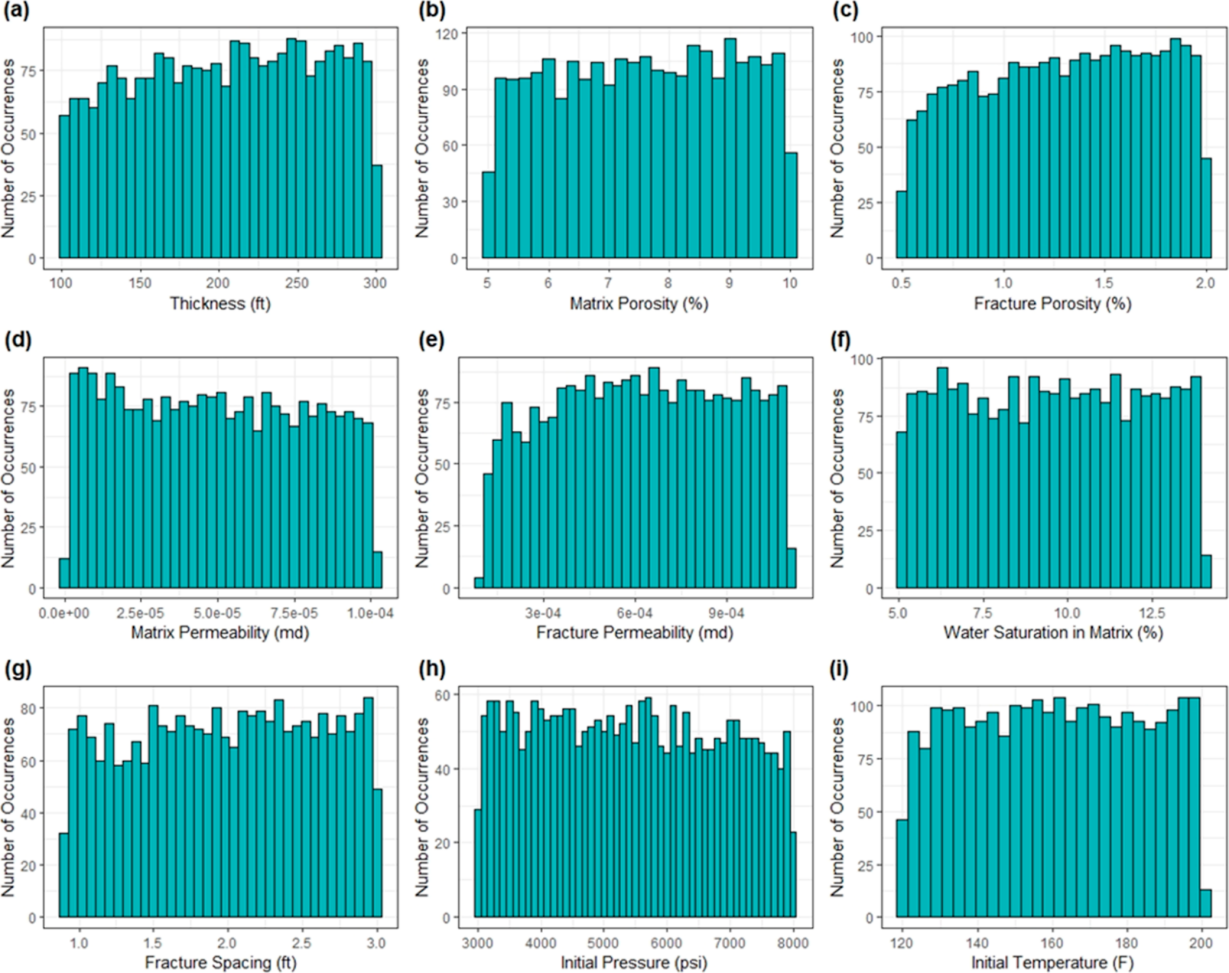
Histograms of reservoir parameters: (a) thickness, (b) matrix porosity, (c) fracture porosity, (d) matrix permeability, (e) fracture permeability, (f) water saturation in the matrix, (g) fracture spacing, (h) initial pressure, and (i) initial temperature.

**Figure 3:**
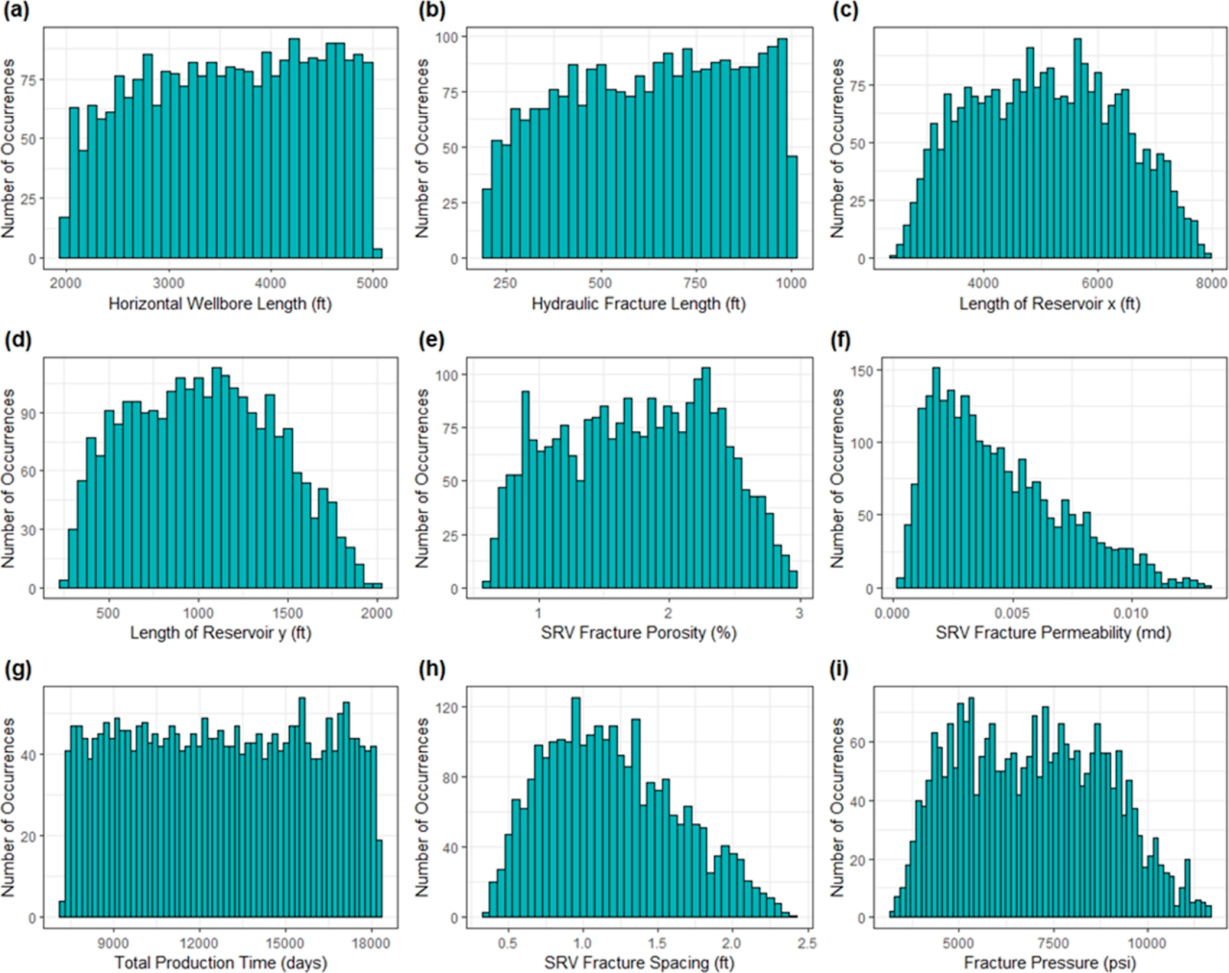
Histograms of operational parameters: (a) horizontal wellbore length, (b) hydraulic fracture length, (c) length of reservoir (*x*), (d) length of reservoir (*y*), (e) SRV fracture porosity, (f) SRV fracture permeability, (g) SRV fracture spacing, (h) total production time, and (i) fracture pressure.

The first step in the analysis involved plotting histograms to understand the data and its distributions. The histograms of all reservoir parameters are analyzed in Figure 2. This analysis shows that reservoir parameter histograms depict a nearly symmetric distribution. This implies that the skewness of the reservoir parameters is approximately zero. In addition, they do not display any type of variability. Similarly, the histograms of all operational parameters are shown in Figure 3. It is observed that most of the operational parameters are nearly symmetric. Except for stimulated reservoir volume fracture permeability (SRV_kf) and stimulated reservoir volume fracture spacing (SRV_xs), other operational parameters do not display any degree of skewness. These histograms clearly show that most sample values are on the left, and the right side of the tail is longer, showing that the histograms are skewed to the right and demonstrate log–normal behavior.

Additionally, in Figure 3f,h**,** stimulated reservoir volume fracture permeability (SRV_kf) and stimulated reservoir volume fracture spacing (SRV_xs) appear to follow a similar behavior, respectively. Because these two variables are required to explain the hydraulic fractures in the SRV zone, thus that is the reason the pattern may be comparable. Ultimately, when comparing reservoir and operational parameters, reservoir parameters do not have any skewness in their histograms. Nonetheless, for operational parameters the variables SRV_kf and SRV_xs to some extent depict a moderately positive skewed behavior.

### Scatterplots

**Figure 4:**
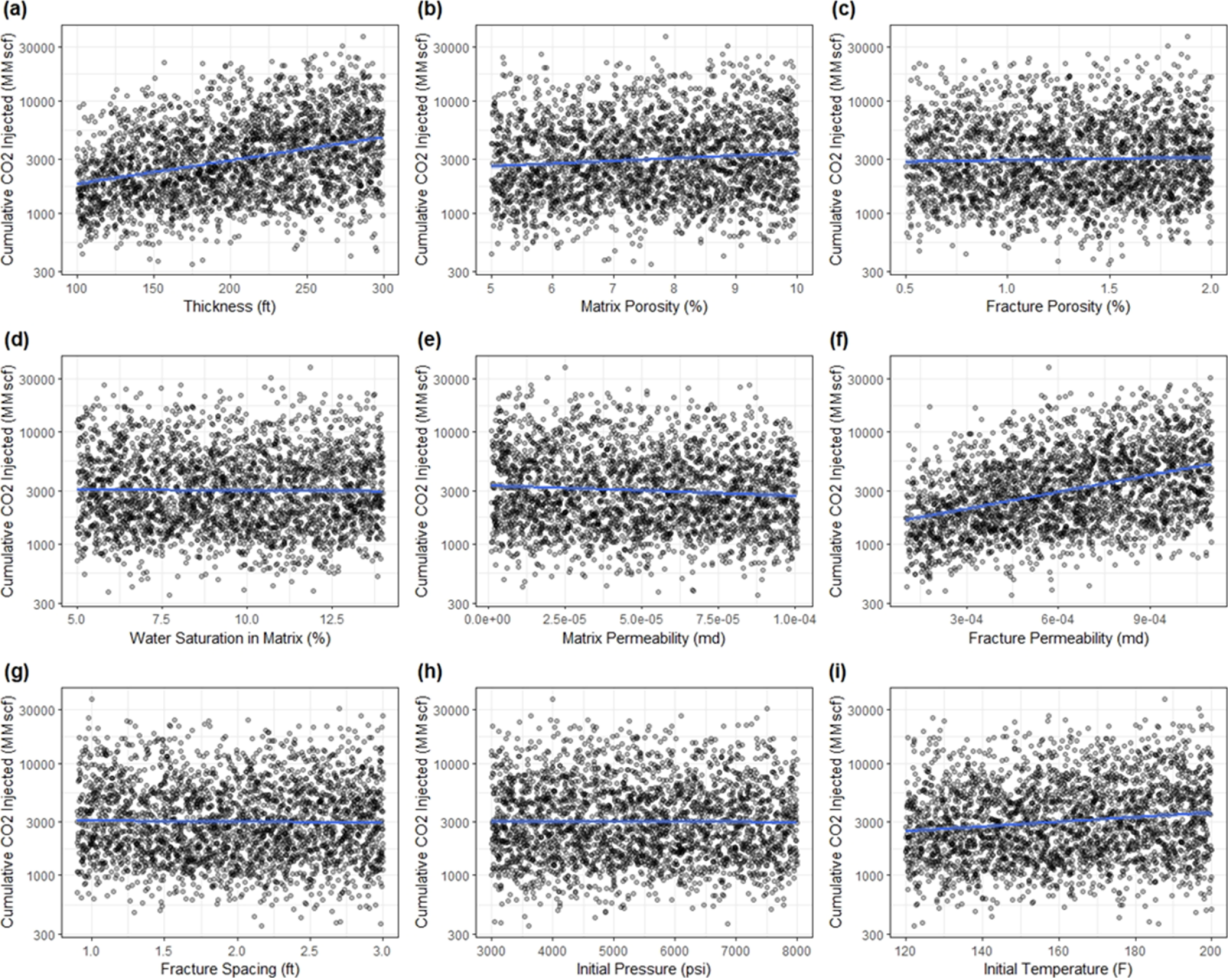
Scatterplots of reservoir parameters vs cumulative CO_2_ injected: (a) thickness, (b) matrix porosity, (c) fracture porosity, (d) matrix permeability, (e) fracture permeability, (f) water saturation in the matrix, (g) fracture spacing, (h) initial pressure, and (i) initial temperature.

**Figure 5:**
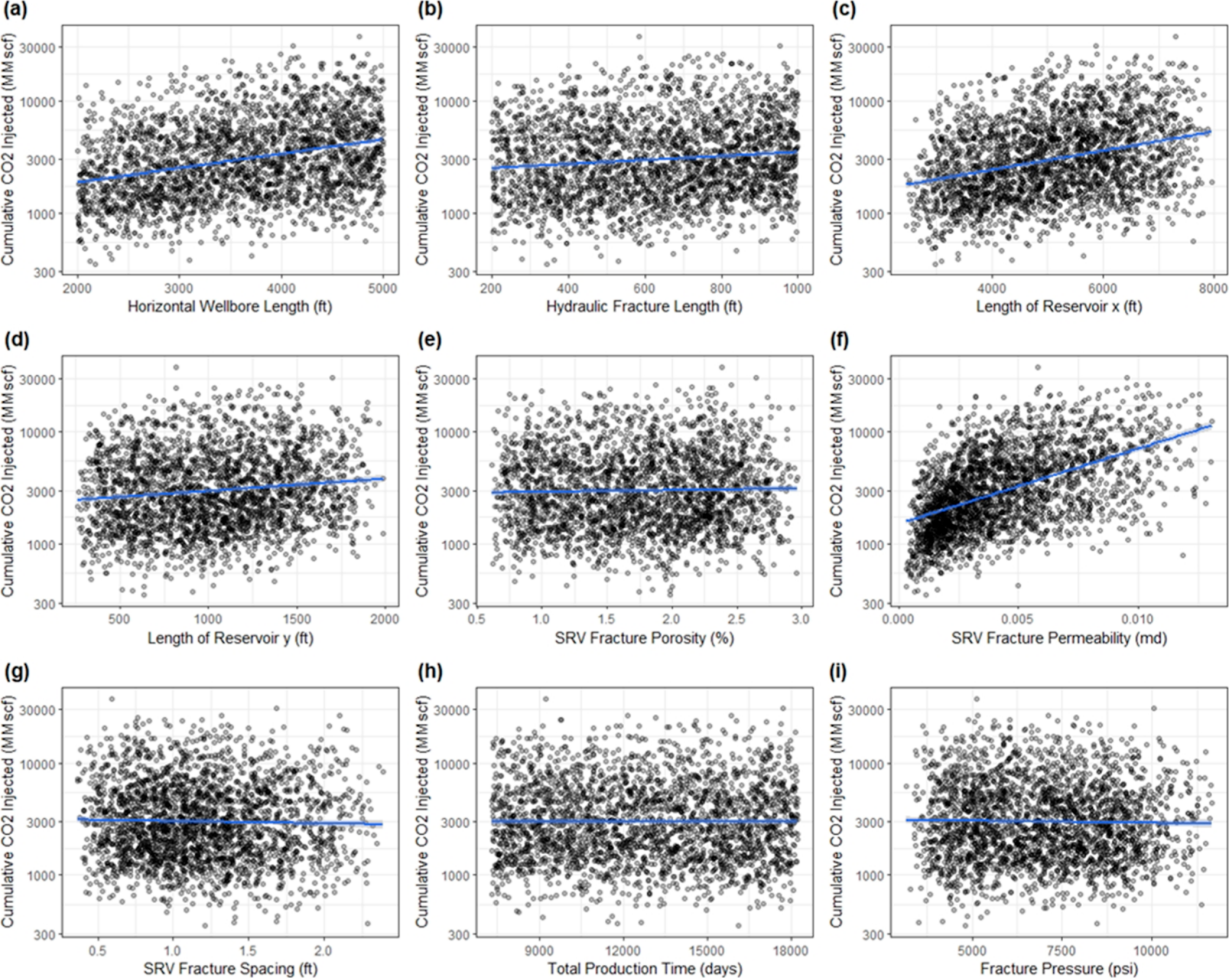
Scatterplots of operational parameters vs cumulative CO_2_ injected: (a) horizontal wellbore length, (b) hydraulic fracture length, (c) length of reservoir (*x*), (d) length of reservoir (*y*), (e) SRV fracture porosity, (f) SRV fracture permeability, (g) SRV fracture spacing, (h) total production time, and (i) fracture pressure.

The second approach in the analysis involved plotting scatterplots in order to understand the relationship between the predictor variables and the response variable. Multiple scatterplots are shown in Figure 4, displaying reservoir parameters against cumulative CO_2_ injected. The lines represent a linear association and trend between the predictor variables and the response variable. In the top-left panel of Figure 4a, a positive linear relationship can be observed between thickness of the reservoir and the cumulative CO_2_ injected. The middle-right panel of Figure 4f shows a modest positive linear trend between the fracture permeability and the cumulative CO_2_ injected. Also, it appears that the cumulative CO_2_ injected has a non-monotonic relationship with the other reservoir parameters. Moreover, scatterplots of operational parameters against cumulative CO_2_ injected are also plotted in Figure 5.

In the top-left panel of Figure 5a, a positive linear trend between the horizontal wellbore length and cumulative CO_2_ injected can be observed. The top-right panel in Figure 5c shows a modest positive linear trend between length of the reservoir in the *x* direction and the cumulative CO_2_ injected. Likewise, the middle-right panel in Figure 5f shows a modest positive linear relationship between the stimulated reservoir volume fracture permeability and the cumulative CO_2_ injected. Stimulated reservoir volume fracture permeability shows a pronounced impact on the cumulative CO_2_ injected. The strength of the linear association between the predictor variables and the response variable will be quantified using the correlation matrix. Lastly, it can be shown that, when comparing reservoir with operational parameters, operational parameters appear to have a greater relevance for the performance metric (cumulative CO_2_ injected) because more operational parameters have a monotonic relation with the performance metric.

### Correlation Matrix

**Figure 6:**
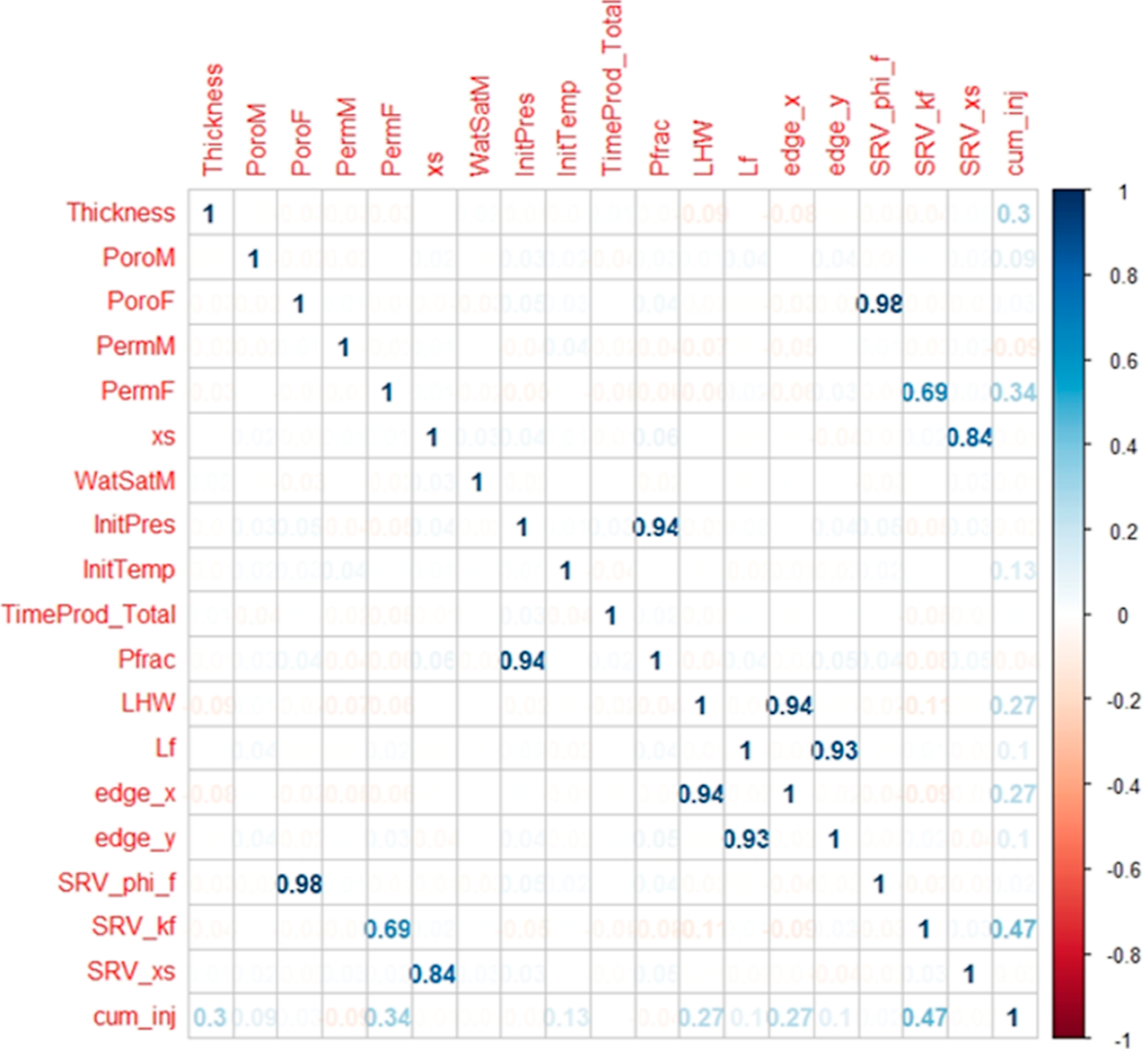
Correlation matrix.

In this study, machine learning algorithms are used to capture the multivariate relationships to predict cumulative CO_2_ injected because no single bivariate relationship provides enough correlation to accurately predict the target variable. The correlation matrix shown in Figure 6 displays the correlation values of all the predictor variables and the response variable. This correlation matrix is important because it gives a general overview of the linear association and relationship between the predictor variables and the main response variable, including among predictor variables. It can be observed in Figure 6 that there is a modest positive correlation between cumulative CO_2_ injected and the stimulated reservoir volume fracture permeability (SRV_kf) with a correlation coefficient (*r*) of 0.47.

This implies that stimulated reservoir volume fracture permeability has a more pronounced influence on the cumulative CO_2_ injected. This analysis reveals that stimulated reservoir volume fracture permeability is an influential parameter to describe the SRV zone in which a large amount of the injected CO_2_ will be stored. Cumulative CO_2_ injected and fracture permeability are positively correlated with a correlation coefficient (*r*) of 0.34. Additionally, cumulative CO_2_ injected is positively correlated to thickness with a correlation coefficient (*r*) of 0.3. Reasonable correlations were found between predictor variables such as fracture porosity (PoroF) and stimulated reservoir volume fracture porosity (SRV_phi_f), length of the reservoir in the *x* direction (edge_x) and horizontal wellbore length (LHW), stimulated reservoir volume fracture spacing (SRV_xs) and fracture spacing (xs), together with fracture pressure (Pfrac) and initial pressure (InitPres). Hence, a significant number of operational parameters have a modest positive association with the cumulative CO_2_ injected. As a result, they are critical to characterize the SRV zone, where practically all the injected CO_2_ will be stored.

### Multiple Linear Regression

After performing exploratory data analysis and understanding our data, the next step was to build predictive models to predict the volume of CO_2_ sequestered. The first predictive modeling tool used was multiple linear regression. Before carrying out multiple linear regression, the data was randomly split into 70% of training set and the remaining 30% was used as a testing set for our accuracy. Cumulative CO_2_ injected was the main response variable that measures the total amount of CO_2_ injected in million standard cubic feet (MMscf). Both reservoir and operational parameters are included in the 18 variables that make up the predictors.

**Figure 7:**
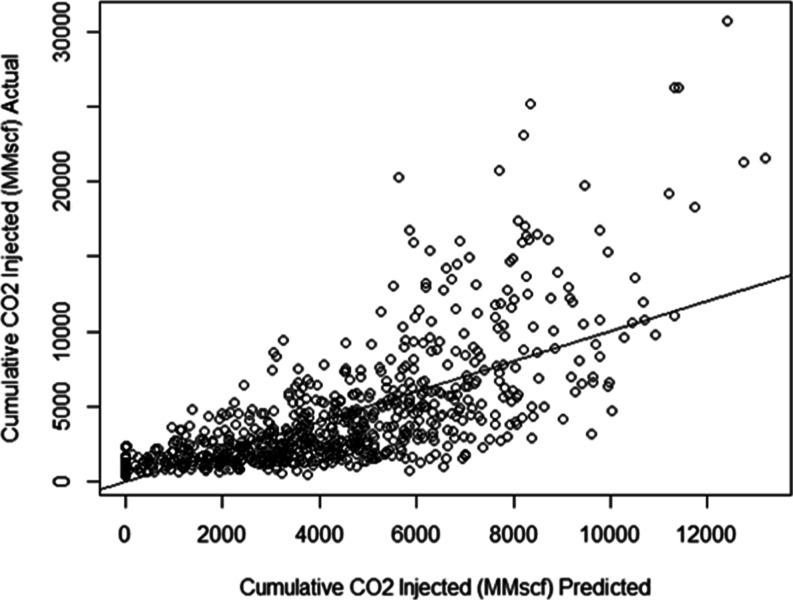
Actual vs predicted cumulative CO_2_ injected for the multiple regression model.

Using the RMSE and *R*^2^ approaches, we evaluated how well the model performed on the full data set of 18 variables. The model was first trained, and the summary of the model is given in Table A1. Based on the summary, the *R*^2^ value is approximately 0.49. The goodness of fit and the variability explained by the 18-variable model are represented by this value of *R*^2^. *R*-squared with an approximate value of 0.49 explains a moderate percentage of the variation in the response variable. Moreover, the model was evaluated and tested by predicting on the remaining 30% of our testing set. Figure 7 shows the cross-plot of the actual versus the predicted values of the cumulative CO_2_ injected. The diagonal black line represents the model fit. The *R*-squared value corresponding to the model fit is approximately 49% which is a moderate fit. This model gives an RMSE value of 2932.6 MMscf. The regression equation for the multiple linear regression model between the response variable and input variables are as follows
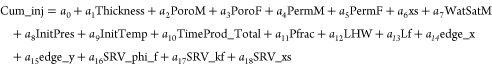
3

**Table A1:** Multiple Linear Regression Summary^a^

*Y*: cumulative CO_2_ injected				
coefficients	estimate	std. error	*t* value	Pr(>|*t*|)
(intercept)	–1.683 × 10^4^	9.036 × 10^2^	–18.625	<2 × 10–^16^ ***
thickness	2.368 × 10^1^	1.143 × 10^0^	20.713	<2 × 10–^16^ ***
PoroM	2.136 × 10^2^	4.452 × 10^1^	4.797	1.75 × 10–^6^ ***
PoroF	9.609 × 10^2^	7.650 × 10^2^	1.256	0.209227
PermM	–6.560 × 10^6^	2.213 × 10^6^	–2.964	0.003076**
PermF	3.905 × 10^5^	3.206 × 10^5^	1.218	0.223421
xs	8.972 × 10^1^	1.980 × 10^2^	0.453	0.650470
WatSatM	–6.271 × 10^0^	2.503 × 10^1^	–0.251	0.802221
InitPres	2.729 × 10–^1^	1.297 × 10–^1^	2.104	0.035518*
InitTemp	2.679 × 10^1^	2.852 × 10^0^	9.396	<2 × 10–^16^***
TimeProd_Total	2.964 × 10–^2^	2.068 × 10–^2^	1.433	0.152024
Pfrac	–2.072 × 10–^1^	9.769 × 10–^2^	–2.121	0.034031*
LHW	7.406 × 10–^1^	2.367 × 10–^1^	3.129	0.001784**
Lf	1.463 × 10^0^	7.903 × 10–^1^	1.851	0.064271
edge_x	5.874 × 10–^1^	1.564 × 10–^1^	3.755	0.000179***
edge_y	1.150 × 10–^1^	4.471 × 10–^1^	0.257	0.797043
SRV_phi_f	–3.349 × 10^2^	5.550 × 10^2^	–0.603	0.546357
SRV_kf	6.858 × 10^5^	3.326 × 10^4^	20.618	<2 × 10–^16^***
SRV_xs	–4.647 × 10^2^	2.720 × 10^2^	–1.708	0.087769

a Residual standard error: 2723 on 1763 degrees of freedom. Multiple *R*-squared: 0.4919 and adjusted *R*-squared: 0.4867. F-statistic: 94.83 on 18 and 1763 DF, and *p*-value: <2.2 × 10^–16^.

**Figure 8:**
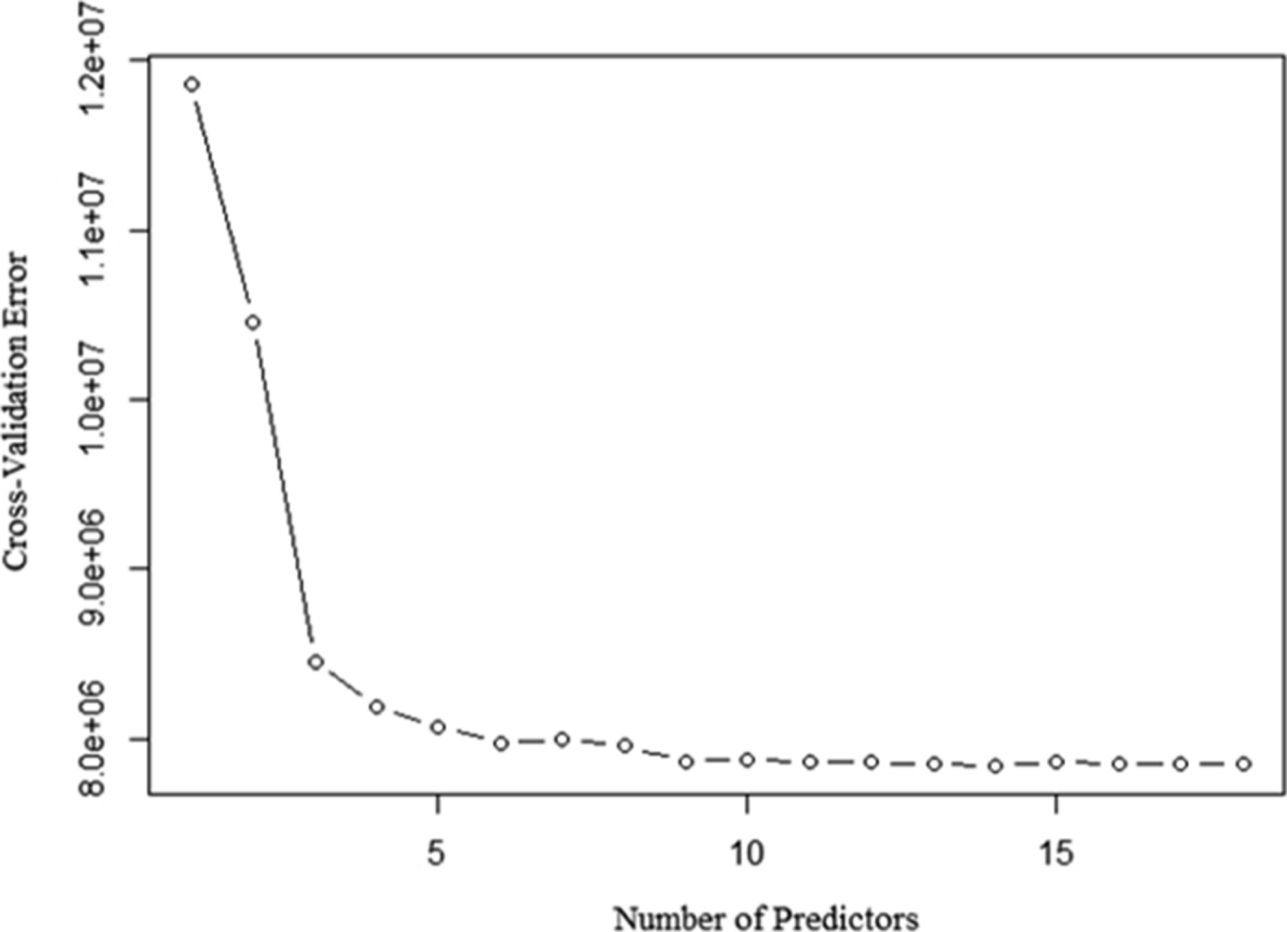
*k*-fold cross-validation method.

The corresponding regression coefficients can be found in Table A1. Following the construction of the full model, we used a cross-validation approach to down-select from the full list of the 18 predictor variables to just those variables which meaningfully contribute to prediction. Figure 8 displays the cross-validation errors on the shale reservoir data set by using *k* = 10 folds. This *k*-fold cross-validation model selection process picks a 14-variable model which has the lowest cross-validation errors.

The regression equations for the multiple linear regression model between the response variable and input variables for the cross-validation method is as follows
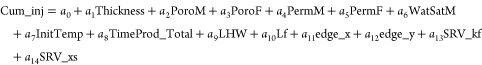
4

The coefficients for the best model of cross-validation can be found in Table A2. Lastly, we build our final multiple linear regression model (Table A3) based on the 14-variable model and check the diagnostic plots to see if there are potential problems and if we will need to perform a transformation.

**Table A2:** Cross-Validation Coefficients

coefficients	value
intercept	–1.676 × 10^4^
thickness	2.435 × 10^1^
PoroM	2.206 × 10^2^
PoroF	5.445 × 10^2^
PermM	–6.982 × 10^6^
PermF	4.056 × 10^5^
WatSatM	–2.797 × 10^1^
InitTemp	2.391 × 10^1^
TimeProd_Total	4.218 × 10–^2^
LHW	9.452 × 10–^1^
Lf	8.247 × 10–^1^
edge_x	4.716 × 10–^1^
edge_y	4.759 × 10–^1^
SRV_kf	7.135 × 10^5^
SRV_xs	–2.821 × 10^2^

**Table A3:** Multiple Linear Regression Summary^a^

*Y*: cumulative CO_2_ injected				
coefficients	estimate	std. error	*t* value	Pr(>|*t*|)
(intercept)	–1.676 × 10^4^	7.450 × 10^2^	–22.502	<2 × 10^–16^***
thickness	2.435 × 10^1^	9.777 × 10^–1^	24.907	<2 × 10^–16^***
PoroM	2.206 × 10^2^	3.844 × 10^1^	5.740	1.06 × 10^–8^***
PoroF	5.445 × 10^2^	1.309 × 10^2^	4.160	3.29 × 10^–5^***
PermM	–6.982 × 10^6^	1.914 × 10^6^	–3.648	0.000269**
PermF	4.056 × 10^5^	2.763 × 10^5^	1.468	0.142205
WatSatM	–2.797 × 10^1^	2.120 × 10^1^	–1.319	0.187150
InitTemp	2.391 × 10^1^	2.416 × 10^0^	9.897	<2 × 10^–16^***
TimeProd_Total	4.218 × 10^–2^	1.759 × 10^–2^	2.398	0.016540 *
LHW	9.452 × 10^–1^	2.016 × 10^–1^	4.688	2.91 × 10^–6^***
Lf	8.247 × 10^–1^	6.693 × 10^–1^	1.232	0.217965
edge_x	4.716 × 10^–1^	1.340 × 10^–1^	3.520	0.000440***
edge_y	4.760 × 10^–1^	3.786 × 10^–1^	1.257	0.208827
SRV_kf	7.135 × 10^5^	2.866 × 10^4^	24.894	<2 × 10^–16^***
SRV_xs	–2.821 × 10^2^	1.266 × 10^2^	–2.229	0.025919 *

a Residual standard error: 2790 on 2532 degrees of freedom. Multiple *R*-squared: 0.49 and adjusted *R*-squared: 0.4857. F-statistic: 172.7 on 14 and 2532 DF, and *p*-value: <2.2 × 10^–16^.

**Figure 9:**
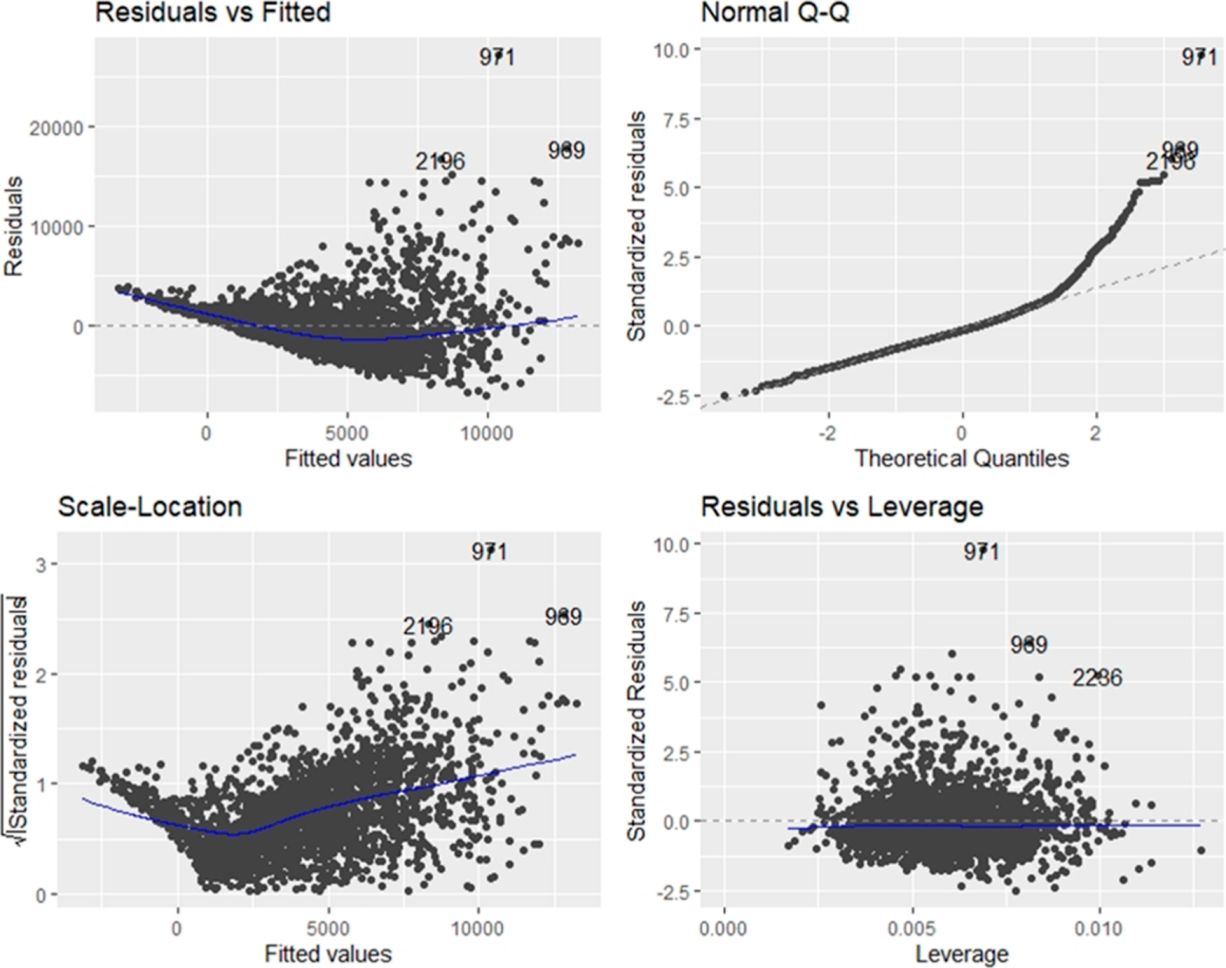
Regression diagnostic plots.

Figure 9 represents the regression diagnostics based on the 14-variable model. The residuals versus fitted plot indicates a pattern which means that there is a problem with the linear model, and we should consider logging our predictors. Furthermore, the normal Q–Q plot does not follow a straight line which suggests there is no normality, and the scale–location plot displays a heteroscedasticity pattern meaning the variances are not constant.

A potential solution to the above problems is to log transform the variables which displayed a high positive skewness based on the visual analysis of the previous histograms and to perform a log transformation to the response variable in order to decrease the problem of heteroscedasticity. This finding highlights why performing an exploratory data analysis was imperative. Because now we can determine which predictors were not normally distributed and we can log transform them. These predictors include SRV_kf and SRV_xs. Table A4 shows the results for the nonlinear transformation of the predictors and the response variable. The *R*-squared metric was used to determine how well a model performed on the data set. We observed a significant improvement on the value of *R*-squared from 49% without transformation to 66% with log transformation.

**Table A4:** Multiple Linear Regression Summary^a^

*Y*: cumulative CO_2_ injected				
coefficients	estimate	std. error	*t* value	Pr(>|*t*|)
(intercept)	7.475 × 10^0^	3.763 × 10–^1^	19.867	<2 × 10–^16^ ***
thickness	5.739 × 10–^3^	1.652 × 10–^4^	34.750	<2 × 10–^16^ ***
PoroM	4.861 × 10–^2^	6.489 × 10–^3^	7.491	9.40 × 10–^14^ ***
PoroF	1.196 × 10^–1^	2.209 × 10^–2^	5.414	6.74 × 10^–8^ ***
PermM	–8.526 × 10^2^	3.229 × 10^2^	–2.641	0.00832 **
PermF	–1.682 × 10^1^	4.826 × 10^1^	–0.349	0.72743
WatSatM	–4.714 × 10^–3^	3.579 × 10^–3^	–1.317	0.18788
InitTemp	5.115 × 10^–3^	4.079 × 10^–4^	12.542	<2 × 10^–16^ ***
TimeProd_Total	1.220 × 10^–5^	2.972 × 10^–6^	4.104	4.19 × 10^–5^ ***
LHW	2.616 × 10^–4^	3.404 × 10^–5^	7.685	2.17 × 10^–14^ ***
Lf	3.086 × 10^–4^	1.134 × 10^–4^	2.722	0.00654 **
edge_x	9.453 × 10^–5^	2.263 × 10^–5^	4.178	3.04 × 10^–5^ ***
log(edge_y)	6.065 × 10^–2^	5.798 × 10^–2^	1.046	0.29563
log(SRV_kf)	7.194 × 10^–1^	1.957 × 10^–2^	36.752	<2 × 10^–16^ ***
log(SRV_xs)	–1.055 × 10^–1^	2.403 × 10^–2^	–4.391	1.17 × 10^–5^ ***

a Residual standard error: 0.4709 on 2532 degrees of freedom. Multiple *R*-squared: 0.6563 and adjusted *R*-squared: 0.6544. F-statistic: 345.3 on 14 and 2532 DF, and *p*-value: < 2.2 × 10^–16^.

### Tree Methods

**Figure 10:**
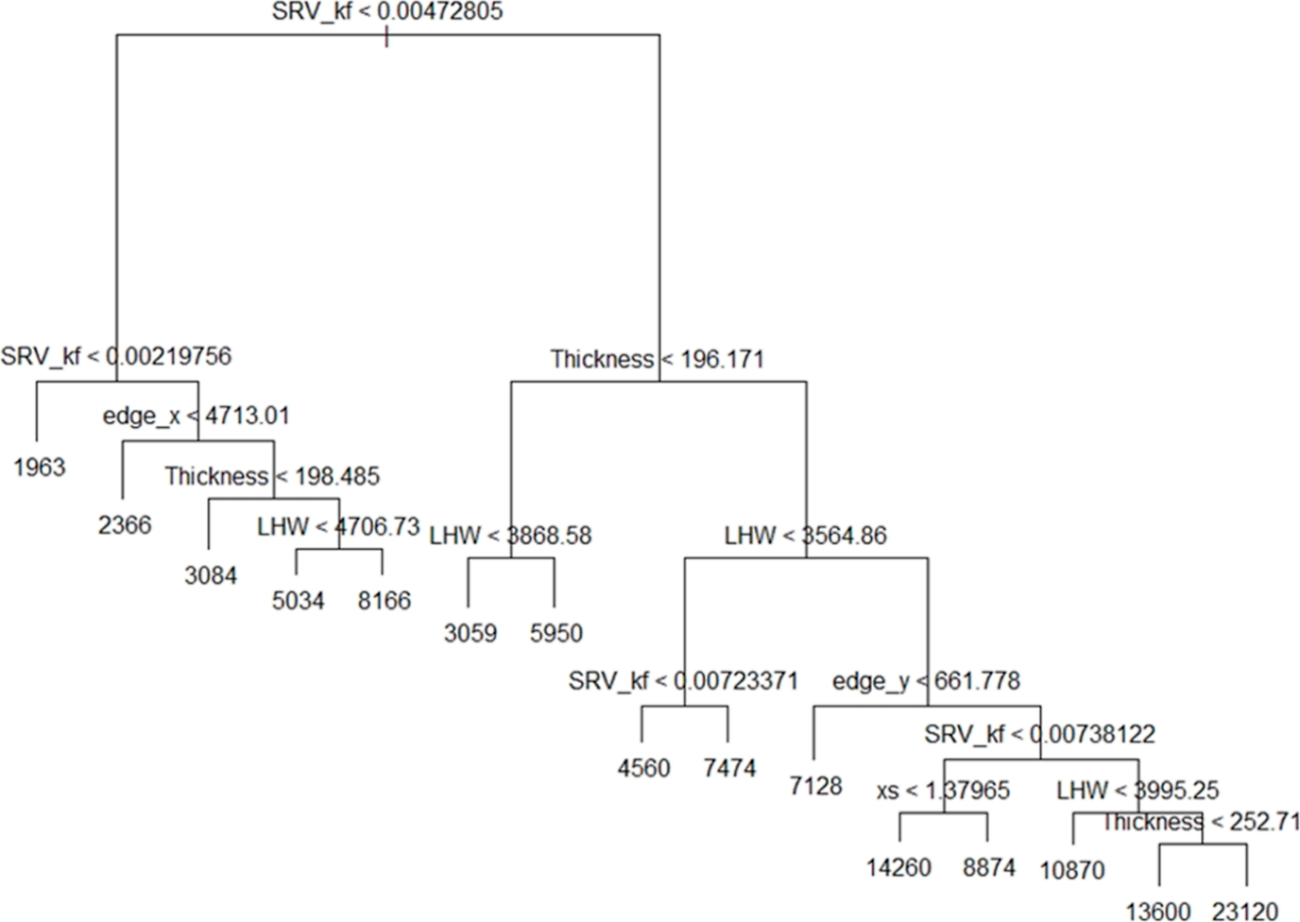
Regression tree.

The model in the previous section, which used a multiple linear regression model, contained assumptions that had to be met and thus could not represent nonlinear behavior without a transformation. Tree-based approaches do not make any assumptions about linearity at the outset, thus they may capture nonlinear behavior and are easily understood. The first tree method utilized was the regression tree method. It can be visualized from Figure 10 that the tree splits the operational and reservoir parameters into 15 regions of predictor space. In addition, only six variables have been used in the construction of the tree. The effectiveness of regression tree can be seen in Figure 10 because it efficiently deduces the most important parameters that are affecting the cumulative CO_2_ injected. These are SRV_kf, thickness, and LHW. Furthermore, this tree can provide an interpretation of how to obtain a high-volume sequestration case. To give an example, when SRV_kf ≥ 0.0047 md, thickness ≤ 196.2 ft, and LHW ≥ 3868.6 ft the predicted cumulative CO_2_ injected volume is 5950 MMscf.

**Figure 11:**
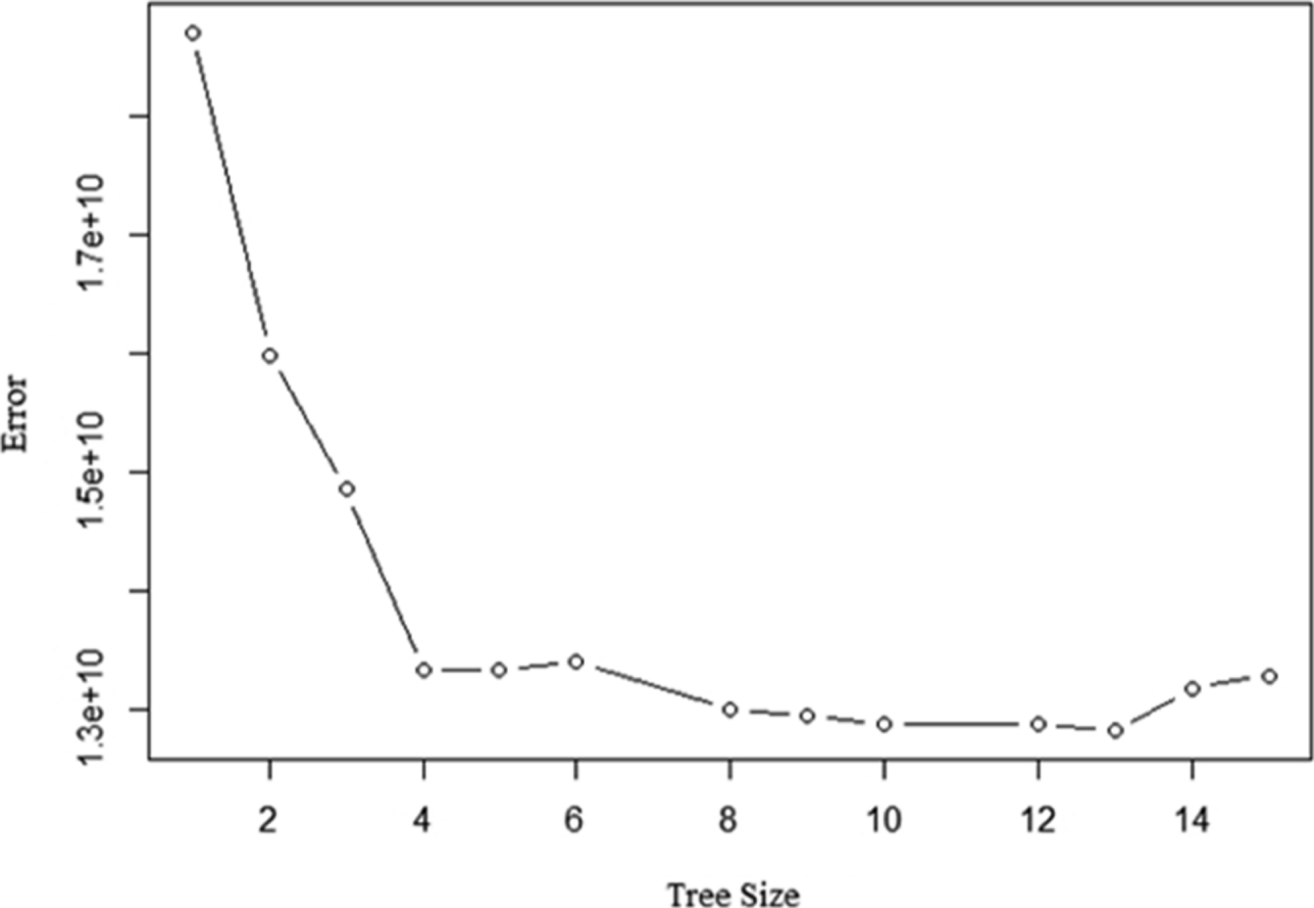
Cross-validation error rate.

**Figure 12:**
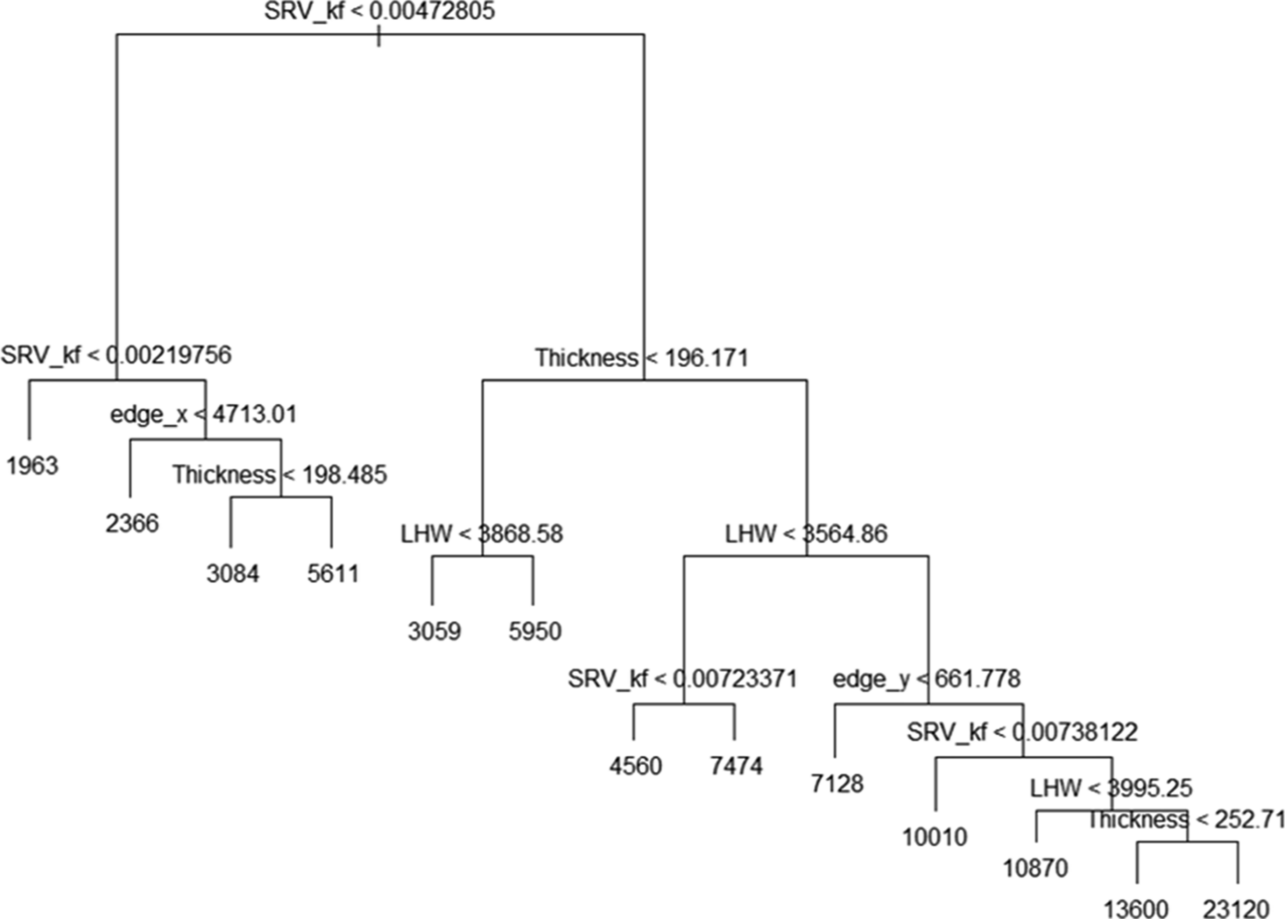
Pruned regression tree.

After fitting the full tree, we used a cross-validation approach to prune the tree. Pruning the tree helps to prevent overfitting and leads to better interpretation. Figure 11 displays the cross-validation error with the tree size (number of terminal nodes) to be considered. As seen in Figure 11, the tree with 13 terminal nodes results in the lowest error rate and therefore we can prune the tree to 13 terminal nodes. The pruned tree with the smallest cross-validation error can be seen in Figure 12.

**Figure 13:**
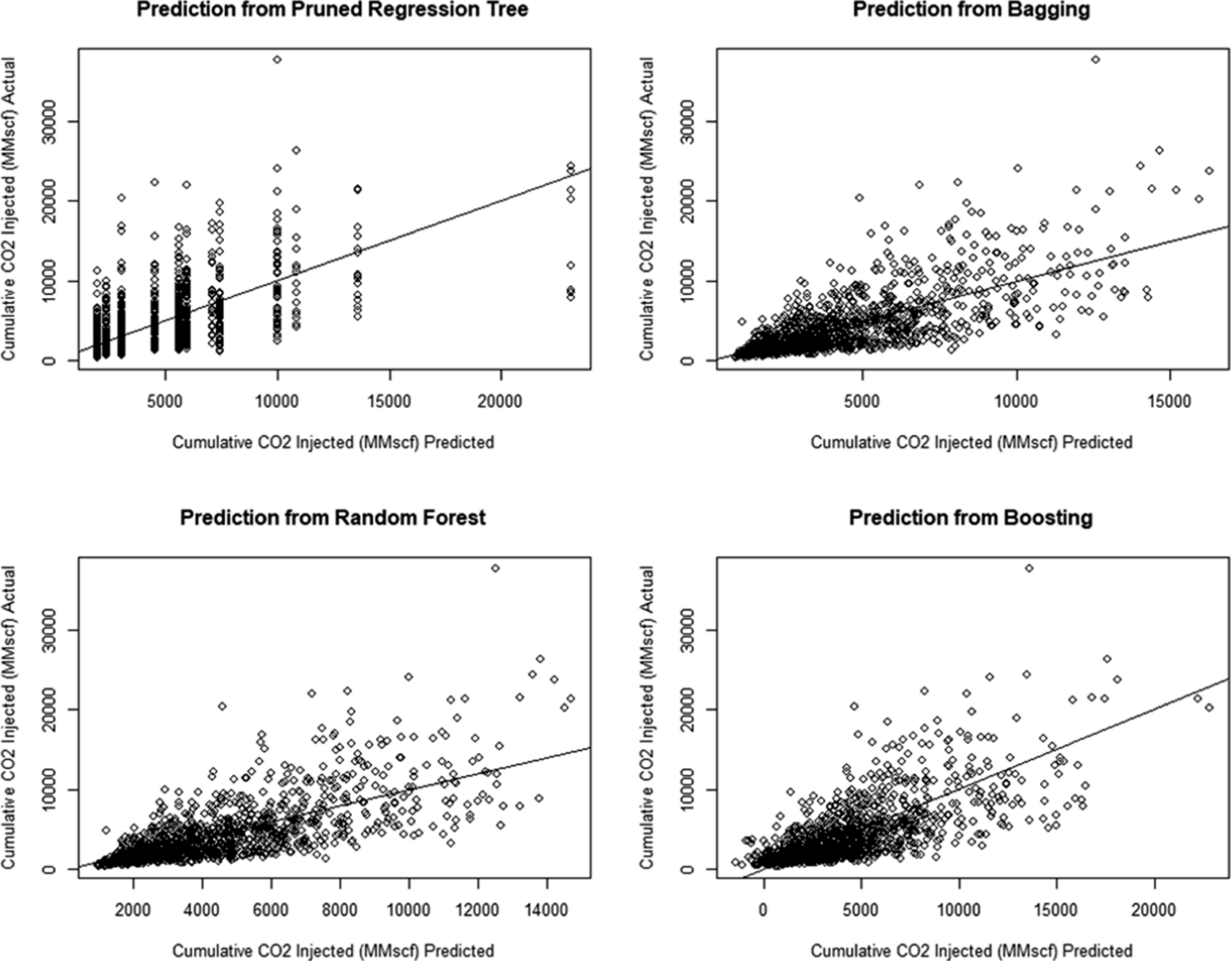
Cross-plots of tree-based methods.

More robust approaches, such as bagging, random forest, and boosting, were applied to improve the results of the prior regression tree. Figure 13 shows cross-plots of all tree-based methods. For the pruned regression tree model, this model gives an MSE of 9.8 × 10^6^ MMscf^2^ and a test RMSE of 3129 MMscf, which is equivalent to 3.13 Bscf. For the bagging model, the test MSE gives a value of 7.3 × 10^6^ MMscf^2^ and a test RMSE of 2707 MMscf and this corresponds to 2.707 Bscf. Moreover, for the random forest model, this model gives an MSE of 7.3 × 10^6^ MMscf^2^ and a test RMSE of 2706 MMscf and this corresponds to 2.706 Bscf.

Lastly, for the boosting model, this model gives an MSE of 7.8 × 10^6^ MMscf^2^ and RMSE 2798 MMscf which corresponds to 2.798 Bscf. Subsequently, a comparison of all machine learning models is reached in order to determine which model is the best for predicting CO_2_ sequestration performance in unconventional shale reservoirs. Random forest surpasses all other machine learning approaches in terms of accuracy. Because of its 2.706 Bscf prediction error, it is the most reliable.

### Variable Importance

**Figure 14:**
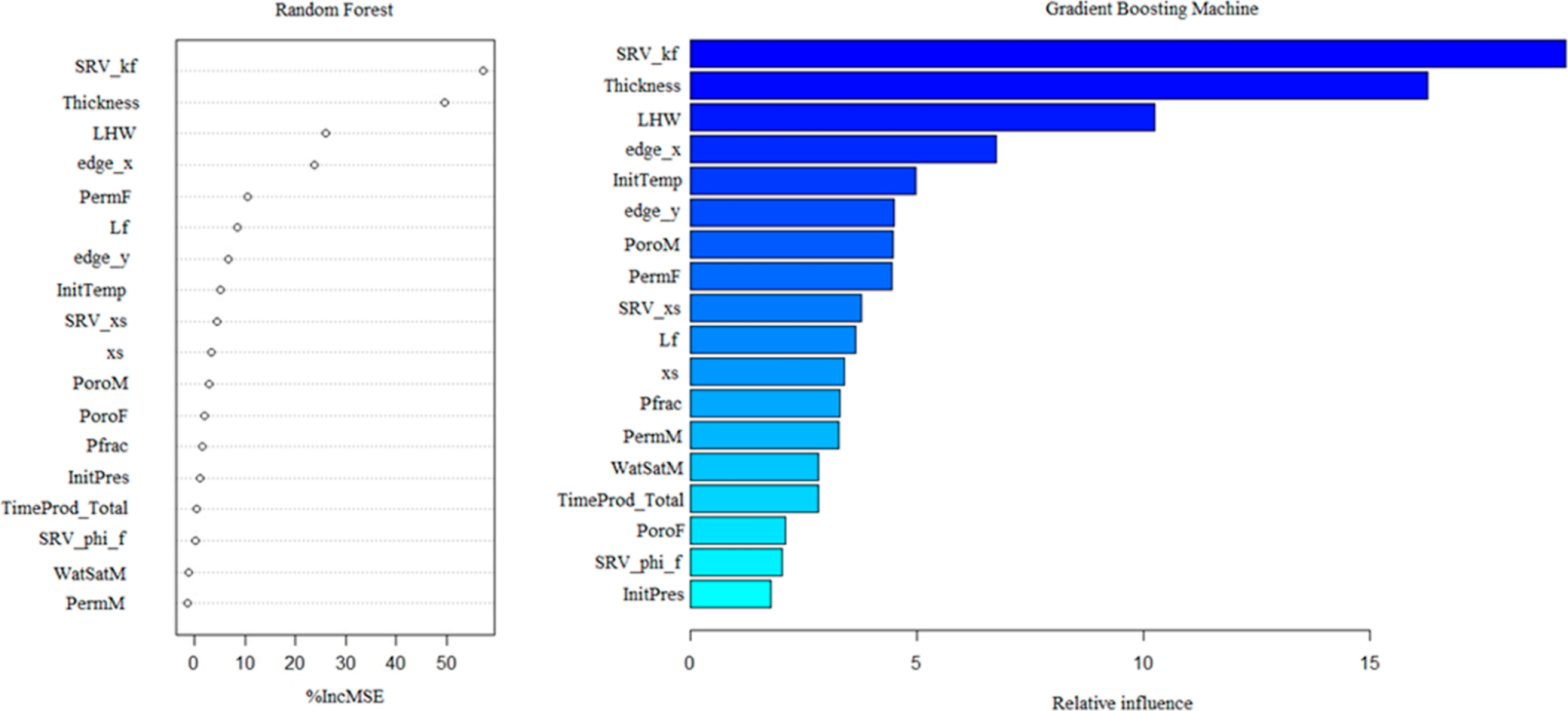
Variable importance for the random forest model (left) and the GBM model (right).

The final goal of this research was to determine the primary drivers of CO_2_ sequestration in unconventional shale gas reservoirs. This procedure is mostly controlled by examining the response variable in numerous predictor variables. Random forest and boosting contain built-in methods for running such a procedure to determine the most influential predictors to support with this. Based on the random forest and boosting approaches, Figure 14 depicts the relevance of each of the 18 predictors for the extensive shale reservoir data set.

When compared to other parameters in the study, these prominent variables have a greater effect on predicting the performance of the CO_2_ sequestration process. It can be observed in Figure 14 that the stimulated reservoir volume fracture permeability (SRV_kf) is the most important predictor and has the greatest influence on the cumulative CO_2_ injected. For both approaches, this variable is the most important because the SRV zone is a part of the reservoir that has been stimulated, and the fracture apertures have developed in size and become more conductive. Hence, the fluid flow and overall mobility will be enhanced.

The next influential variable for both methods is thickness. The thickness of the reservoir has a significant impact on reserve capability. Thus, it is an important parameter for the performance of CO_2_ sequestration process. Length of the horizontal wellbore was also highly ranked. This is mainly because the well intersects highly conductive fractures, which would help in the formation of CH_4_ for injection of CO_2_. A lengthy horizontal wellbore would increase the contact area with the SRV zone, which would have a significant impact on the well’s productivity index. Length of the reservoir in the x direction is also influential. The remaining parameters have a smaller impact.

## Conclusions

In this paper, we implemented a data analytics-based investigation and machine learning methods on an extensive shale reservoir data set. The key objectives included ascertaining patterns and features within the shale reservoir data set and developing predictive models based on regression and tree-based machine learning methods. Moreover, this article provides insights into the relationship between reservoir parameters, operational parameters, and the volume of CO_2_ sequestered as well as the most prominent variables that affect the volume of CO_2_ sequestered.

Based on the data analytics investigation that was carried out, it was concluded that:

- When compared with reservoir parameters, operational parameters appear to have a high skewness based on their histograms.
- It can be shown that, when comparing reservoir parameters with operational parameters, operational parameters appear to have a greater impact on the response variable because more operational parameters have a linear association with the response variable.
- The cumulative CO_2_ injected has a modest positive correlation with a range of operational parameters.

Based on the machine learning models that were developed, it was concluded that:.

- Regression trees are simple to learn and can rank which parameters have the most effect on the volume of CO_2_ sequestered.
- Because it had the lowest prediction error, random forest has the highest predictive ability among the machine learning methods.
- Random forest and boosting models can be used for identifying the most important variables affecting CO_2_ sequestration performance.
- Stimulated reservoir volume fracture permeability is the most influential parameter of CO_2_ sequestration performance.
- Based on variable importance methods, we can conclude that operational parameters are paramount for the performance of CO_2_ sequestration.

## Appendix A

Tables A1–A4.
